# Graft materials and regenerative strategies in secondary alveolar bone grafting: A scoping review of randomized clinical trials

**DOI:** 10.1016/j.jobcr.2026.101508

**Published:** 2026-08-04

**Authors:** Alfan Maulana Erdiansyah, Husni Mubarak, Yossy Yoanita Ariestiana, Andi Tajrin

**Affiliations:** Department of Oral and Maxillofacial Surgery, Faculty of Dentistry, Hasanuddin University, Makassar, Indonesia

**Keywords:** Secondary alveolar bone grafting, Alveolar cleft, Cleft lip and palate, Graft material, Regenerative strategies, Scoping review

## Abstract

**Background:**

Secondary alveolar bone grafting (SABG) places bone in the alveolar cleft, crucial for managing cleft lip and palate. SABG restores ridge continuity, aids tooth eruption, and stabilizes the maxilla. Autogenous iliac crest grafting is the standard treatment option. However, varied evidence on donor-site morbidity, graft resorption, and outcomes prompts exploring alternative grafts and adjuncts.

**Objective:**

To systematically map and critically synthesize randomized controlled trial (RCT) evidence on graft materials, adjunctive biologics, and outcome assessment strategies in SABG, and to identify methodological gaps that limit translational decision-making.

**Methods:**

A comprehensive search was performed across PubMed, Scopus, Web of Science, and Embase for RCTs published in the last decade that evaluated graft materials and biologic adjuncts in SABG. Data on graft materials, adjunctive biologics, surgical strategies, outcome assessment methods, and clinical outcomes were extracted and synthesized narratively.

**Results:**

Of 408 records identified, 27 RCTs met the eligibility criteria. Most studies used autogenous iliac crest grafting. Adjunctive strategies included platelet concentrates, cell-based therapies, barrier membranes, graft substitutes, and tissue-engineered constructs. Some studies reported improvements in early healing, intraoperative handling, or bone density. Radiographic assessments were primarily CT and CBCT-based. Outcome measures and reporting approaches varied across studies. Most studies reported short-term outcomes, with limited long-term follow-up data available.

**Conclusions:**

Across the 27 RCTs, autogenous iliac crest bone remained the main graft and comparator, with no adjunct or alternative showing consistent superiority in bone volume, density, or resorption. Adjunct benefits were context-dependent, while heterogeneity in outcomes, imaging, and follow-up limited cross-study comparability.

## 1 Introduction

Orofacial clefts are among the most common craniofacial birth defects, with cleft lip and/or palate affecting approximately 1 in 700 live births worldwide. Cleft lip and palate (CLP), the combined phenotype, has a reported global prevalence of about 0.45 per 1000 live births.^1^ Alveolar clefts disrupt the maxillary arch, leading to impaired tooth eruption, poor periodontal support, oronasal fistula, and nasal base instability. These issues impact speech, chewing, and facial symmetry. Restoring alveolar continuity is critical in cleft care.^2^

Secondary alveolar bone grafting (SABG) is the standard procedure for repairing alveolar clefts.^2^ It is usually performed during mixed dentition to support canine eruption and stabilize the maxillary arch. Autogenous cancellous bone from the anterior iliac crest, the gold standard for alveolar bone grafting because it provides osteogenic, osteoinductive, and osteoconductive benefits, along with fast revascularization and clinical success in alveolar cleft repair.^3^ It is effective in restoring bone continuity. However, drawbacks include donor-site morbidity such as postoperative pain, gait disturbance, scarring, and complications, as well as concerns about graft resorption and long-term stability.^4^

To address these limitations, research has studied alternative autogenous sites, bone substitutes, biologic adjuncts such as platelet-rich plasma (PRP) and platelet-rich fibrin (PRF), and cell-based and tissue-engineered methods involving bone marrow aspirate concentrate (BMAC), stromal vascular fraction (SVF), and stem cell scaffolds. Advances in imaging, mainly cone-beam computed tomography (CBCT), have made outcome assessment more precise and three-dimensional.

Despite extensive research, the evidence base remains fragmented. RCTs evaluating graft materials and adjunctive treatments show highly variable outcomes. Results for bone volume, density, and clinical success are inconsistent. There is also substantial variation in study design, graft composition, surgical techniques, imaging protocols, and outcome measures. Studies use non-standardized volumetric calculations, density assessments, and radiographic scoring systems.^5^ Most studies have follow-up periods of only 3–12 months. This may not capture outcomes such as tooth eruption through the graft, orthodontic movement, and long-term stability.^6^^,^^7^

Systematic reviews have tried to evaluate specific interventions or adjunctive strategies. Most focus on comparing effectiveness, but they face the same heterogeneity and lack of standardized outcomes. Thus, there is no clear overview of how graft materials, adjunctive techniques, and outcome assessments are studied in randomized trials. This makes it hard to find research patterns, gaps, and future priorities.

Given this complexity, a scoping review is suitable. Scoping reviews systematically map the extent, range, and main features of evidence in emerging, heterogeneous fields. They help identify key concepts, gaps, and trends without being limited to pooled effect estimates.

The aim of this scoping review is to map RCTs on graft materials and regenerative strategies in SABG. Specifically, this review will: (1) identify the types of graft materials and adjunctive approaches studied; (2) describe surgical and regenerative strategies used; (3) examine outcome measures and imaging methods; (4) summarize reported outcomes; and (5) identify research gaps for future studies and better decision-making.

## 2 Methods

### 2.1 Protocol and registration

This scoping review used the Joanna Briggs Institute (JBI) methodology (Peters et al., 2020). Reporting followed the PRISMA-ScR guidelines (Tricco et al., 2018). The review protocol was registered with the Open Science Framework (OSF) prior to the start (DOI: 10.17605/OSF.IO/WZQ86). Any protocol deviations were documented and reported.

### 2.2 Eligibility criteria

#### 2.2.1 Inclusion criteria

Studies were included if they fulfilled these criteria: (1) Population — patients with cleft lip and palate needing alveolar bone grafting; (2) Concept — evaluation of bone grafting procedures, including graft materials and adjuncts; (3) Context — clinical studies from any country or healthcare setting; (4) Study design — Randomized clinical trials; (5) Outcomes — studies reporting bone volume, density, bone fill percentage, clinical outcome, complication.

#### 2.2.2 Exclusion criteria

Studies were excluded if they: (1) lacked radiographic outcome assessment; (2) had a follow-up duration of less than three months; (3) were review articles, editorials, or conference abstracts without full-text availability; or (4) were published in languages other than English without an available translation. Excluding non-English studies may introduce language bias and limit generalizability, as relevant research published in other languages could have offered additional perspectives or different results. This potential bias should be considered when interpreting the findings of this review.

#### 2.2.3 Information sources

A comprehensive search was conducted across four electronic databases: PubMed, Scopus, Web of Science, and Embase. The search included publications from January 2016 to March 2026. The reference lists of included studies and relevant systematic reviews were also searched manually. This ten-year window was selected deliberately to capture the contemporary evidence base. It corresponds to the period in which CBCT became the predominant tool for three-dimensional volumetric assessment of alveolar grafts, and in which regenerative adjuncts began to be evaluated in randomized trials. Foundational studies on autogenous iliac crest grafting and earlier graft materials published before 2016 have already been synthesized in previous reviews. The search was confined to these four electronic databases; no supplementary hand-searching of reference lists or citation tracking was undertaken.

#### 2.2.4 Search strategy

A two-step search strategy was employed following JBI recommendations. First, a limited initial search was conducted in PubMed and Scopus to identify relevant articles and index terms. Second, a comprehensive search was performed across all four databases using a developed strategy combining keywords and MeSH/EMTREE terms related to: (1) cleft lip and palate (e.g., 'cleft lip,' 'cleft palate,' 'alveolar cleft,' 'orofacial cleft'); (2) alveolar bone grafting (e.g., 'alveolar bone grafting,' 'secondary alveolar bone grafting,' 'bone graft'); and (3) study design (e.g., 'randomized controlled trial,' 'clinical trial'). Third, the reference lists of all included studies were screened for additional eligible studies. The complete search strings and the total yield per database are presented in Table S1.

#### 2.2.5 Study selection

All search results were imported into Mendeley, a reference management software, for deduplication. The remaining records were imported into Rayyan.ai for systematic screening. Two independent reviewers screened titles and abstracts against the eligibility criteria. Full-text articles were retrieved for potentially eligible studies and independently assessed by two reviewers. Disagreements at any stage were resolved through discussion, with a third reviewer available for arbitration. The study selection process is documented in the PRISMA-ScR flow diagram.

#### 2.2.6 Data extraction

A standardized extraction form was developed a priori and piloted on five randomly selected studies. Extracted data included: (1) study characteristics (author, year, country, design, sample size); (2) participant characteristics (age, sex, cleft type, dentition stage); (3) intervention details (graft material, adjunctive biomaterials); (4) outcome measures (assessment methods, follow-up duration, primary and secondary outcomes); and (5) key findings (bone formation metrics, resorption rates, complications). Two reviewers independently extracted data, and discrepancies were resolved through discussion. Corresponding authors were contacted for clarification as necessary.

#### 2.2.7 Data analysis and synthesis

Given the heterogeneity in interventions, outcome measures, and study designs, a narrative synthesis approach was employed following JBI and PRISMA-ScR guidance. Frequencies and percentages were calculated for categorical variables. Study characteristics, interventions, and outcomes were organized in thematic tables and synthesized narratively by category: graft materials and sources, adjunctive biomaterials, surgical techniques, outcome assessment methods, and clinical outcomes. No meta-analysis was performed, consistent with the scoping review's purpose of mapping rather than synthesizing comparative effectiveness.

## 3 Results

### 3.1 Study selection

**Fig. 1:**
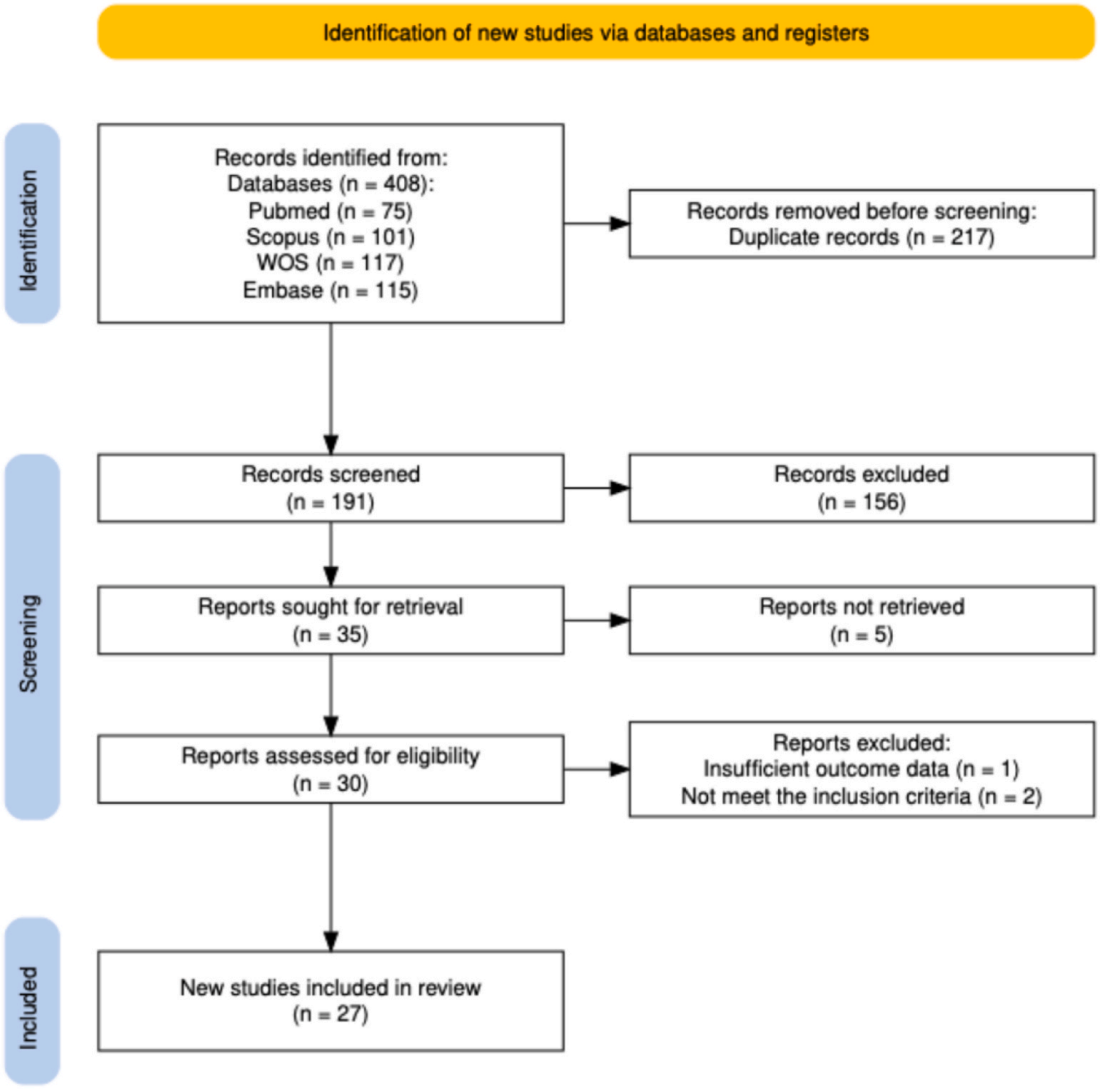
Prisma Flow Diagram.

The systematic search across four electronic databases yielded 408 records, including 75 from PubMed, 101 from Scopus, 117 from Web of Science, and 115 from Embase. After removal of 217 duplicate records, 191 unique studies remained for title and abstract screening. Of these, 156 records were excluded because they did not meet the eligibility criteria, primarily due to non-randomized study designs, irrelevant interventions, non-alveolar cleft populations, or the absence of radiographic outcome assessment. Full-text articles were sought for 35 potentially eligible studies; however, five reports could not be retrieved for eligibility assessment because no full-text report was available: three were clinical-trial registry records without an associated full-text publication, and two were conference abstracts whose full reports could not be obtained. The remaining 30 full-text articles were assessed for eligibility, and three studies were excluded due to insufficient outcome data (n = 1), failure to meet the inclusion criteria (n = 2). Ultimately, 27 randomized controlled trials were included in the review. The study selection process is summarized in Fig. 1.

### 3.2 Study characteristics

The 27 included studies were published between 2016 and 2026 and represented a range of surgical centers, primarily in Egypt, Thailand, India, China, and Middle East. The pooled study population comprised 777 patients. Most patients had non-syndromic unilateral complete cleft lip and palate (UCLP), whereas a smaller but important subset had bilateral cleft lip and palate (BCLP). All the included studies were randomized controlled trials, indicating a relatively high level of methodological rigor within the contemporary SABG literature.

Most studies have focused on patients with unilateral alveolar clefts (n = 21), while six investigations included a mix of unilateral and bilateral clefts. Interventions were predominantly performed during the mixed dentition phase (ages 7–12 years), which corresponds to the standard timing for secondary grafting prior to maxillary canine eruption. A smaller subset addressed late secondary grafting in patients with permanent dentition.

Autogenous bone harvested from the anterior iliac crest was the predominant graft material used in 26 of the 27 studies, either as the primary intervention or as a comparator. Alternative donor sites, such as the mandibular symphysis and maxillary cortical bone, have been evaluated in a limited number of trials. Several studies have investigated adjunctive biologic or regenerative strategies, including platelet-rich fibrin (PRF) and its derivatives, platelet-rich plasma (PRP), bone marrow aspirate concentrate (BMAC), guided bone regeneration (GBR) using acellular dermal matrix or collagen membranes, and, more recently, stromal vascular fraction (SVF) and tissue-engineered constructs.

Radiographic evaluation was primarily conducted using (CBCT), which enabled a three-dimensional volumetric assessment of graft incorporation, bone density, and defect fill. Advanced image-processing software (e.g., Mimics, Romexis, 3D Slicer, Simplant) was frequently employed for quantitative analysis. The principal outcome domains included newly formed bone volume, bone resorption rate, bone density (expressed in Hounsfield units), and alveolar arch continuity. Secondary outcomes encompassed oronasal fistula closure, periodontal parameters, donor-site morbidity, and postoperative pain.

Follow-up durations were generally short to intermediate, with most studies reporting outcomes at 3–12 months postoperatively. Only one study extended the follow-up beyond two years, and one study reported a two-year follow-up, whereas all remaining studies reported follow-up periods of 3–12 months. The detailed study characteristics are presented in Table 1.

**Table 1:** Study characteristics.

Author (Year)	Study Design	n	Population	Intervention	Graft Material(s)	Follow-up	Key Findings
Takemaru et al. (2016)^5^	Randomized controlled trial	15	Age range: NR, Mean ± SD age: Group I: 8.4 ± 1.0 years, Group II: 8.6 ± 1.2 years, M/F subjects: NR	Cancellous iliac bone alone vs + hydroxyapatite–collagen composite (HA/Col)	Autogenous iliac cancellous; autogenous iliac + HA/Col	1, 6, 12 mo	HA/Col was absorbed and replaced by autogenous bone over time; lower intraoperative blood loss and PCA use in HA/Col group; comparable 12-mo bone fill.
Xiao et al. (2016)^6^	Randomized controlled trial	60	Age range: NR, Mean ± SD age: NR, M/F subjects: NR	Iliac crest bone ± guided bone regeneration (GBR) with ADM film	Autogenous iliac ± ADM membrane (GBR)	3 mo	Bone resorption rate significantly lower in GBR group (31.69% vs 36.50%, p < 0.05).
Movahedian Attar et al. (2017)^7^	Randomized controlled trial	20	Age range: 8 to 14 years, Mean ± SD age: 9.7 ± 1.7 years, M/F subjects: 11/9	Chin symphysis + allograft (Cenobone) + L-PRF vs iliac crest graft	Autogenous chin + allograft + L-PRF; autogenous iliac	12 mo	Bone regeneration 69.5% vs 73.8%; operative time shorter in chin group (43 vs 76 min); quicker gait recovery.
Shirani et al. (2017)^8^	Randomized controlled trial	30	Age range: 8-27 years, Mean ± SD age: 15.0 ± 5.7 years, M/F subjects: 17/13	Autogenous iliac + PRGF vs FDBA + PRGF	Autogenous iliac corticocancellous + PRGF; FDBA + PRGF	6 mo	Higher volume retention ratio in iliac + PRGF group (V2/V1 = 0.7 vs 0.5); no oronasal fistulae in either group; both groups showed significant volume decrease.
Huang (2018)^9^	Randomized controlled trial	20	Age range: 8-25 years, Mean ± SD age: NR, M/F subjects: NR	Iliac cancellous + CGF vs + ADM membrane	Autogenous iliac + CGF or ADM	6 mo	Resorption not significantly different; density improvement rate significantly higher in CGF group (61.62% vs 27.05%).
Omidkhoda et al. (2018)^10^	Randomized controlled trial	10	Age range: 9-12 years, Mean ± SD age: 11.3 ± 0.83 years, M/F subjects: 6/4	Autogenous anterior iliac crest bone graft ± PRF	Autogenous iliac ± PRF	3 mo	No significant difference in thickness change, bone height reduction, or bone loss between PRF and control groups.
Alnajjar et al. (2020)^11^	Randomized controlled trial	20	Age range 9–13 yr, Mean age NR; M/F: 11/9	Autogenous iliac chips vs bovine xenograft + I-PRF	Autogenous iliac; bovine xenograft + I-PRF	6 mo	Comparable clinical and radiographic success; no significant difference in bone density between groups (xenograft + I-PRF: 520 ± 170 HU; autograft: 425 ± 120 HU).
Chen et al. (2020)^12^	Randomized controlled trial	40	Age range: 8-13 years, Mean ± SD age: NR, M/F subjects: 31/9	Iliac cancellous bone graft ± PRP	Autogenous iliac ± PRP	6 mo	BF% 42.54% vs 46.97%; all cases repaired fistulae and formed osseous bridge; no significant difference between groups.
Chu et al. (2020)^13^	Randomized controlled trial	44	Age range: 8.54 to 12.3 years, Mean ± SD age: NR, M/F subjects: 28/16	SABG vs SABG with extensive gingivoperiosteoplasty (EGPP)	Autogenous iliac cancellous (SABG alone vs SABG + EGPP)	12 mo	No significant difference in residual bone defect volume at 1 yr between subgroups; most patients classified as Bergland Type I; canine eruption rate not significantly different between groups.
Kadry et al. (2021)^14^	Randomized controlled trial	10	Age range: 9-11 years, Mean ± SD age: NR, M/F subjects: 5/5	BM-MSC–seeded collagen scaffold vs iliac crest autograft	Tissue-engineered construct (BM-MSCs + collagen); autogenous iliac	6, 12 mo	No significant difference in bone volume (p = 0.91 at 6 mo; p = 0.994 at 12 mo) or density; tissue-engineered graft density increased significantly from 6 to 12 mo (p = 0.048).
Thanasut et al. (2021)^15^	Randomized controlled trial	15	Age range: 8-14 years old, Mean ± SD age: NR, M/F subjects: 9/4	PCBM from iliac crest ± PRF	Autogenous iliac PCBM ± PRF	6 mo	No significant difference in regenerated bone volume or density between PRF and control; Chelsea scale results similar; PRF improved graft particle handling.
Rao et al. (2021)^16^	Randomized controlled trial	30	Age range: 7 to 15 years, Mean ± SD age: NR, M/F subjects: 18/12	Iliac cancellous bone + (I + A)-PRF vs iliac bone alone	Autogenous iliac + (I + A)-PRF vs iliac alone	3, 6 mo	PRF combination showed better Bergland scores and greater bone volume at 3 and 6 mo; improved periodontal status in both groups.
Attar et al. (2022)^17^	Randomized controlled trial	22	Age range: NR, Mean ± SD age: 9.7 ± 1.7 years, M/F subjects: 10/12.	Chin symphysis corticocancellous bone + allograft vs anterior iliac crest	Autogenous chin + allograft (Cenobone); autogenous iliac	12 mo	Bone formation 76.9% vs 77.0%; no significant difference; operative time shorter in chin group (40 vs 76 min); all patients with normal gait day 1 in chin group.
Kumar et al., (2022)^18^	Randomized controlled trial	20	Age range: 7 and 16 years, Mean ± SD age: NR, M/F subjects: Group I: 4/6, Group II: 7/3	Iliac cancellous bone vs bovine DMBM + collagen membrane	Autogenous iliac; DMBM (Osseograft) + collagen membrane	6 mo; mean 63 mo	Comparable bone uptake at 6 mo (∼70%); no significant difference at 63 mo; donor-site morbidity in iliac group; higher cost in DMBM group.
Datarkar et al. (2023)^19^	Randomized controlled trial	20	Age range: 7 to 15 years, Mean ± SD age: NR, M/F subjects: 13/7	Cancellous vs cortico-cancellous iliac graft	Autogenous cancellous; cortico-cancellous iliac	6 mo	Faster bridge formation in cancellous group; slower resorption in cortico-cancellous group (16.87% vs 18.74%).
Attar (2023)^20^	Randomized controlled trial	20	Age range: 8 to 12 years, Mean ± SD age: NR, M/F subjects: 11/9	Autologous iliac crest bone graft ± fibrin glue	Autogenous iliac ± fibrin glue	4 mo (1 wk, 1 mo, 4 mo)	Mean bone formation 66.7% vs 76.2% (p = 0.055); fibrin glue may reduce dehiscence; no significant volumetric difference.
Lai et al. (2023)^21^	Randomized controlled trial	49	Age range: 7-18 years, Mean ± SD age: 11.20 ± 2.76 (ICIC) and 11.08 ± 2.56 (OCIC), M/F subjects: 16/9 (ICIC) and 17/7 (OCIC)	Oblique vs iliac cortical-cancellous block harvesting (OCIC vs ICIC)	Autogenous iliac cortico-cancellous block (two harvest techniques)	6 mo	Lower pain scores in OCIC group on POD1; LFCN injury in 3 ICIC patients; duck gait in OCIC vs circle gait in ICIC; no significant difference in pain duration, claudication, or scar.
Omara et al. (2023)^22^	Randomized controlled trial	10	Age range: NR, Mean ± SD age: 9.96 ± 2.84 years (control) and 10.02 ± 2.03 years (study), M/F subjects: 6/4	Cancellous iliac bone vs mineralized plasmatic matrix (MPM)	Autogenous iliac cancellous; MPM from iliac cancellous bone	6 mo	Control group showed significantly greater volume, labio-palatal width, and height reduction compared with MPM group. Control: 48.91% vs. MPM: 19.64% reduction
Shabaan et al. (2023)^23^	Randomized controlled trial	36	Age range: 8 to 11 years, Mean ± SD age: NR, M/F subjects: 17/19	Iliac bone graft + BMAC vs iliac bone graft alone	Autogenous iliac + BMAC; autogenous iliac	12 mo	BMAC + iliac group: significantly superior bone volume, density, and reduced bone loss ratio at 6 and 12 mo; all defects filled; bone loss 4% vs 8%.
Aldaghir et al. (2024)^24^	Randomized controlled trial	72	Age range: 9–11 years; Mean ± SD age: NR; M/F: NR	Iliac cancellous vs maxillary cortical bone chips	Autogenous (iliac cancellous; maxillary cortical)	3, 6 mo	Mean volume: 0.81 vs 0.74 cm^3^; no significant difference in vertical bone loss (p = 0.547), fistula development (p = 0.074), or arch continuity (p = 0.058).
Desai et al. (2024)^25^	Randomized controlled trial	30	Age range: 8 to 15 years, Mean ± SD age: 13.43 ± 2.71, M/F subjects: 18/12	Iliac bone + BMAC + PRF vs iliac bone + BMAC	Autogenous iliac + BMAC ± PRF	12 mo	Bone regeneration 75% (BMAC + PRF) vs 70% (BMAC only); resorption 25% vs 30%; faster wound healing in PRF group.
Eid et al. (2024)^26^	Randomized controlled trial	36	Age range: 7-12 years, Mean ± SD age: NR, M/F subjects: 22/14	PRF + chin bone vs collagen membrane + chin bone vs chin bone alone	Autogenous chin ± PRF or collagen membrane	9 mo	PRF and collagen membrane groups showed enhanced bone density vs control; significant between-group differences at 9 mo.
Parmar et al. (2024)^27^	Randomized controlled trial	20	Age range: 7 to 9 years, Mean ± SD age: 8 years, M/F subjects: 13/7	Cancellous particulate vs cortico-cancellous block iliac graft	Autogenous cancellous; cortico-cancellous block (iliac)	6 mo	100% arch continuity in both groups; faster bone bridge formation in cancellous group; volume ratio 94.94% vs 89.06%.
Puvvada (2025)^28^	Randomized controlled trial	20	Age range: 8-25 years, Mean ± SD age: 11.2 ± 5.4 years, M/F subjects: 8/12	Autologous cortico-cancellous bone + PRP vs + PRP + Bio-Oss	Autogenous + PRP; autogenous + PRP + Bio-Oss	2 yr (1 wk, 1, 3, 6 mo, 1, 2 yr)	Group B (+Bio-Oss) showed significantly higher bone density at 6 mo and 1 yr; greater bone regeneration in Group B; no significant difference in wound healing or initial pain.
Raafat et al. (2025)^29^	Randomized controlled trial	18	Age range: 7-13 years old, Mean ± SD age: Control group: 9.59 ± 2.00 years, Intervention group: 9.33 ± 1.98 years, M/F subjects: Control group: 5/4, Intervention group: 4/5	Double iliac corticocancellous block + cancellous particulates vs conventional cancellous graft	Autogenous iliac (double block + particulates vs cancellous)	9 mo	Intervention group: significantly higher graft width, height, and volume at 9 mo; control group had significantly greater volume reduction (51% vs 14.4%); longer operative time in intervention group.
Yadav et al. (2025)^30^	Randomized controlled trial	75	Age range: 7 to 20 years, Mean ± SD age: Group 1: 12.8 ± 2.3, Group 2: 13.1 ± 2.5, Group 3: 13.0 ± 2.1, M/F subjects: Group 1: 15/10, Group 2: 14/11, Group 3: 16/9	Autologous bone graft alone vs + PRP vs + PRF	Autogenous (alone, + PRP, + PRF)	6 mo	Highest bone density and Bergland Grade I success in ABG + PRF group at 6 mo; lower VAS pain scores in PRF group for 7 days; low complication rates across all groups.
Kurimori et al. (2026)^31^	Randomized controlled trial	15	Age range: NR, Mean ± SD age: Control group: 17.35 years, Experimental group: 27.6 years; M/F subjects: 10/5	Iliac crest bone graft ± stromal vascular fraction (SVF)	Autogenous iliac ± SVF	6 mo	Comparable bone development between groups; SVF did not significantly improve volumetric outcomes; graft volume correlated with cleft-side improvement (R = 0.78).

Abbreviations: mo = months; yr = years; RCT = randomized controlled trial; PCBM = particulate cancellous bone and marrow; DMBM = demineralized bone matrix; FDBA = freeze-dried bone allograft; PRF = platelet-rich fibrin; PRP = platelet-rich plasma; PRGF = plasma rich in growth factors; I-PRF = injectable PRF; L-PRF = leukocyte-PRF; (I + A)-PRF = injectable and advanced PRF; BMAC = bone marrow aspirate concentrate; SVF = stromal vascular fraction; BM-MSCs = bone marrow–derived mesenchymal stem cells; HA/Col = hydroxyapatite–collagen composite; ADM = acellular dermal matrix; GBR = guided bone regeneration; MPM = mineralized plasmatic matrix; EGPP = extensive gingivoperiosteoplasty; BF% = bone formation percentage; VAS = visual analogue scale; POD1 = postoperative day 1; LFCN = lateral femoral cutaneous nerve; OCIC = oblique cortical-cancellous iliac crest; ICIC = inner cortico-cancellous iliac crest; HU = Hounsfield units.

### 3.3 Graft materials used in alveolar cleft repair

All 27 included studies evaluated bone grafting interventions for alveolar cleft repair. Autogenous cancellous bone harvested from the iliac crest was used as the reference or control material in most trials and was applied either as the sole grafting material or as the comparator arm.

When corticocancellous iliac crest bone was compared with freeze–dried bone allograft (FDBA), a higher 6-month volume retention ratio was observed in the autograft group (V2/V1 = 0.7 vs. 0.5)^8^. Defect fill of approximately 70% at 6 months, with no significant differences in alveolar height scores, was reported when iliac crest bone was compared with bovine demineralized bone matrix (DMBM); comparable findings were observed at approximately 5-year follow-up.^18^

Xenograft materials have also been investigated. Comparable 6-month bone density and clinical outcomes were reported when bovine xenograft granules combined with injectable platelet-rich fibrin (i-PRF) were compared with iliac crest bone chips.^11^ Greater bone density and regeneration at 6 and 12 months were reported when a composite graft comprising autologous bone, Bio-Oss xenograft, and PRP was compared with autologous bone plus PRP alone.^28^

Tissue-engineered approaches were evaluated in several trials. No significant differences in new bone volume or density at 6 and 12 months were observed between bone marrow–derived mesenchymal stem cell (BM-MSC)-seeded collagen scaffolds and iliac crest autografts.^14^

Alternative autogenous donor sites were also assessed. Mandibular symphysis bone, used alone or combined with iliac crest bone or allograft, demonstrated favorable bone regeneration rates compared to iliac crest grafts. Reduced donor site morbidity, absence of gait disturbance, and shorter operative time were reported with chin bone harvesting.^7^^,^^17^^,^^26^

The distribution of graft materials and adjunctive strategies across the included studies is summarized in Table 2.

**Table 2:** Classification of graft materials and adjunctive strategies across included studies.

Treatment Strategy	Base Graft	Platelet Concentrate	Stem Cell Therapy	Biomaterial Substitute	Membrane	Study Reporting(s)
Autogenous Bone Graft	Iliac cancellous bone	-	-	-	-	^7^^,^^11^^,^^12^^,^^14^^,^^15^^,^^18^^,^^19^^,^^22^^,^^24^^,^^27^
	Iliac cortico-cancellous bone	-	-	-	-	^17^^,^^19^, ^20^, ^21^^,^^27^^,^^29^^,^^31^
	Mandibular symphysis bone	-	-	-	-	^26^
Autogenous Bone + Platelet Concentrates	Iliac cortico-cancellous bone	PRP	-	-	-	^28^
		PRGF	-	-	-	^8^
	Iliac cancellous bone	PRF	-	-	-	^10^^,^^15^^,^^30^
		PRP	-	-	-	^30^
		CGF	-	-	-	^9^
		A-PRF + I-PRF	-	-	-	^16^
	Mandibular symphysis bone	PRF	-	-	-	^26^
	Mandibular symphysis bone	L-PRF	-	Cenobone allograft	-	^7^
	Maxillary cortical bone	PRF	-	-	-	^24^
Autogenous Bone + Stem Cell Therapy	Iliac cancellous bone	-	BMAC	-	Collagen membrane (Bio-Gide®)	^23^
		PRF	BMAC	-	-	^25^
		SVF	-	-	-	^31^
Autogenous Bone + Biomaterials Substitutes	Iliac cancellous bone	-	-	HA/Col	-	^5^
	Iliac cortico-cancellous bone	PRP	-	DBBM (Bio-Oss®)	-	^28^
	Mandibular symphysis bone		-	Allogenic bone graft (CenoBone®)	-	^17^
Autogenous Bone + Barrier Membrane	Iliac cortico-cancellous bone	-	-	-	Fibrin glue (Evicel®)	^20^
	Iliac cancellous bone	-	-	-	ADM membrane	^6^^,^^9^
	Mandibular symphysis bone	-	-	-	Collagen membrane (Colla-D)	^26^
Xenograft-based Grafting + Platelet concentrate	Bovine Xenograft granules	I-PRF	-	-	-	^11^
Xenograft-based Grafting + Barrier membrane	DMBM	-	-	-	Collagen membrane (Healguide)	^18^
Allograft-based Grafting + Platelet concentrate	FDBA	PRGF	-	-	-	^8^
Tissue Engineering	Collagen xenogenic scaffold (Osteovit®)	-	BM-MSCs	-	-	^14^

Abbreviations: PRF = platelet-rich fibrin; PRP = platelet-rich plasma; CGF = concentrated growth factor; A-PRF = advanced platelet-rich fibrin; PRGF = plasma rich in growth factors; I-PRF = injectable PRF; L-PRF = leukocyte-PRF; (I + A)-PRF = injectable and advanced PRF; BMAC = bone marrow aspirate concentrate; SVF = stromal vascular fraction; BM-MSCs = bone marrow–derived mesenchymal stem cells; HA/Col = hydroxyapatite–collagen composite; DBBM = deproteinized bovine bone mineral; ADM = acellular dermal matrix; FDBA = freeze-dried bone allograft; DMBM = demineralized bone matrix.

### 3.4 Adjunctive therapies

Various biologic and biomaterial adjuncts have been evaluated across the included studies. Platelet concentrates, including PRF, PRP, and their derivatives, were the most frequently investigated adjunctive strategies; however, the findings were inconsistent. Advanced PRF formulations demonstrated measurable advantages in bone density and complication reduction.^16^^,^^30^ The mineralized plasmatic matrix (MPM) substantially reduced graft volume loss compared with conventional particulate bone (19.64% vs. 48.91%),^22^ and PRGF favored autogenous over allogeneic bone pairings in terms of volume retention (V2/V1:0.70 vs. 0.50).^8^ In contrast, conventional PRF and PRP used as simple graft admixtures generally failed to demonstrate statistically significant benefits over autogenous bone grafting alone.^10^^,^^12^^,^^15^ Stem cell augmentation using BMAC showed the most consistent regenerative potential among adjunctive approaches, with two independent trials reporting superior bone volume, density, and resorption outcomes compared with conventional iliac grafting.^23^^,^^25^ By contrast, a tissue-engineered bone marrow–derived mesenchymal stem cell (BM-MSC)/collagen construct achieved results comparable to autogenous grafting without donor-site morbidity,^14^ whereas SVF did not provide a significant additive benefit.^31^ Barrier membranes, including acellular dermal matrix (ADM), collagen membranes, PRF membranes, and fibrin glue, tended to reduce early graft resorption and improve bone density in several studies. This included significantly lower resorption in ADM-guided bone regeneration compared with non-GBR approaches (31.69% vs. 36.50%; p = 0.017)^6^ and the absence of oronasal fistula recurrence in membrane-treated groups.^26^ These findings suggest a possible membrane-class effect mediated by the physical exclusion of soft-tissue ingrowth. Biomaterial substitutes appeared to address donor-site morbidity more directly than biologic augmentation. Mandibular symphysis bone combined with allograft achieved bone regeneration rates comparable to iliac crest grafting (69.5%–76.9% vs. 73.8%–77.0%),^7^^,^^17^ and DMBM combined with a collagen membrane maintained comparable bone outcomes to autogenous grafting at a mean 63-month follow-up while eliminating donor-site morbidity.^18^ In addition, xenograft combined with PRF showed favorable early bone formation at 6 months.^11^ Overall, BMAC appears to be the most promising augmentation strategy for maximizing bone regeneration, whereas advanced PRF formulations may offer a relatively low-cost approach to improving bone density and wound healing. Barrier membranes may support early graft preservation and soft-tissue integrity, while graft extenders paired with local autogenous harvest offer a plausible strategy for reducing iliac donor-site morbidity without compromising osseous outcomes.

### 3.5 Radiographical measurement methods

Radiographic assessment methods varied substantially across the included studies, with differences in the imaging modality, measurement type, analytical technique, and software platform. For clarity, these methods were categorized into six domains. The specific methods and tools used are listed in Table 3.

**Table 3:** Radiographic assessment domains, methods, and software tools across included studies.

Domain	Measurement Type	Method/Technique	Software/Tool	Studies Reporting(s)
3D Volumetric	**CBCT volumetric analysis**	Standard CBCT volumetric assessment	*NR*	^7^^,^^15^^,^^17^^,^^24^^,^^28^
		volumetric assessment	*Planmeca Romexis/Romexis Viewer 5.0 (Planmeca)*	^19^^,^^20^^,^^23^^,^^27^
		Manual 3D volumetric assessment	*3D Slicer*	^22^^,^^29^
		Semi-automated 3D reconstruction	*Mimics 17.0 (Materialise)*	^9^
		Cavalieri stereological principle	*NR*	^18^
		Area summation of 1-mm axial CBCT cuts; V1 and V2 ratio	*AutoCAD 2010*	^8^
		Automated 3D volumetric assessment	*Simplant 11.04*	^6^
	**CT volumetric analysis**	3D reconstruction with Boolean subtraction	*Mimics 15.01 (Materialise)*	^14^
		CAE with automated calculation of DV, FV, and BF%	*Mimics 16.0 + Geomagic Studio 2013*	^12^
		Multi-planar reconstruction (MPR) for volumetric analysis	*Syngovia (Siemens Healthcare GmbH, Version VB30)*	^25^
		3D volumetric reconstruction with multiplanar reconstruction (MPR)	*Blender*	^31^
		Volume measurement with CAE	*NS*	^5^
		Serial volume measurement	*NS*	^13^
Density Assessment	**CT density (HU)**	Densitometric analysis using HU	*Mimics 15.01 (Materialise)*	^14^
			*NS*	^26^
			*Blender (open-source)*	^31^
	**CBCT-derived density (HU/gray value)**	CBCT density measured in HU	*Vatech*	^11^
		Density improvement rate (%) in CBCT-derived HU	*Mimics 17.0 (Materialise)*	^9^
		CBCT density measured in Hounsfield units	*Romexis software package*	^10^
		Relative bone density assessed via CBCT	*ImageJ software*	^15^
		Bone density (BD) measured in HU via the Annotations Measure Rectangle tool.	*Planmeca Promax 3D classic/Romexis (Planmeca)*	^23^
		Bone density (HU) assessed via Dentascan	*Dentascan* (GE Health Care Pvt Ltd.)	^28^
Linear/Dimensional	**Height/width/thickness**	height and thickness measured in millimetres	Romexis software package	^10^
		vertical height and width measured	*Syngovia (Siemens Healthcare GmbH, Version VB30)*	^25^
	**Labio-palatal width**	cross-sectional labio-palatal graft width measured	*3D Slicer (open-source)*	^22^
			*Atomica.ai®*	^29^
	**Vertical bone height**	height measured from nasal floor to alveolar crest	*Atomica.ai®*	^29^
Radiographic Scoring Systems	**Bone position**	Chelsea Scale	*ImageJ*	^15^
	**Radiographic grading**	Abyholm 4-point scale	*-*	^24^
		Bergland classification	*-*	^18^
			-	^13^
			-	^30^
			*-*	^16^
2D Radiographic Methods	**Periapical**	Periapical radiographs with Bergland classification	-	^16^
	**Periapical**	Periapical radiographs used for bone density assessment with Chelsea scale	*ImageJ*	^15^
	**IOPA/panoramic/occlusal**	Intraoral periapical, maxillary occlusal, and panoramic radiographs as primary radiographic modality with Bergland classification	*-*	^30^
Composite Approaches	**CBCT + density + scoring**	CBCT volumetric analysis (Cavalieri principle/planimetry) combined with CBCT-derived HU density measurement and modified vertical/horizontal bone scoring tool		^18^
	**CBCT + periapical radiograph**	CBCT for bone volume (%) and relative bone; periapical radiographs for Chelsea scale classification		^15^

Abbreviations: DV = defect volume; FV = final volume; BF% = bone formation percentage; HU = Hounsfield units; CBCT = cone-beam computed tomography; CT = computed tomography; 3D = three-dimensional; CAE = computer-aided engineering; MPR = multiplanar reconstruction; NR = not reported; NS = not specified; IOPA = intraoral periapical.

Most of the studies included used three-dimensional radiographic imaging to quantitatively assess bone outcomes, with cone-beam computed tomography (CBCT) being the predominant modality. CBCT-based volumetric analyses were conducted using a heterogeneous range of software platforms, including Planmeca Romexis Viewer 5.0,^19^^,^^20^^,^^23^^,^^27^ 3D Slicer,^22^^,^^29^ Mimics 17.0^9^, AutoCAD 2010 through sequential area summation of 1-mm axial cuts,^8^ and Simplant 11.04^6^. One study used the Cavalieri stereological principle without dedicated software,^18^ and several studies reported CBCT-based volumetric outcomes without specifying the software platform used.^7^^,^^15^^,^^24^^,^^28^ Conventional CT-based volumetric quantification has also been reported using Mimics 15.01 with Boolean subtraction for three-dimensional reconstruction,^14^ Mimics 16.0 combined with Geomagic Studio 2013 for computer-aided engineering analysis of defect and formed bone volumes,^12^ Syngovia software for multiplanar reconstruction,^25^ Blender for open-source three-dimensional reconstruction,^31^ and computer-aided engineering approaches without software specification.^5^^,^^13^ Bone density was concurrently quantified in Hounsfield units (HU) in a substantial proportion of studies, derived either from conventional CT^14^^,^^26^^,^^31^ or from CBCT gray values converted to HU. The latter approach used platforms including Vatech,^11^ Mimics 17.0 ^9^, Romexis,^10^ ImageJ,^15^ Planmeca Promax 3D Classic with Romexis,^23^ and Dentascan.^28^

In addition to volumetric and densitometric assessments, a subset of studies incorporated linear dimensional measurements and standardized radiographic scoring systems as complementary outcome tools. Linear measurements of graft height, thickness, and labio-palatal width were performed using Romexis,^10^ Syngovia,^25^ 3D Slicer,^22^ and Atomica.ai®.^29^ Among radiographic scoring instruments, the Bergland classification was the most frequently applied 5, 6, 7^,^^31^, followed by the Abyholm four-point vertical bone height scale^24^ and the Chelsea scale for assessing bone position.^15^ Two-dimensional radiographic modalities, including intraoral periapical, maxillary occlusal, and panoramic radiographs, were used as either primary or supplementary assessment tools in a minority of studies.^15^^,^^16^^,^^30^ This represents a methodological limitation acknowledged by the respective authors, given the lower volumetric accuracy of planar imaging compared with CBCT. Composite radiographic approaches integrating volumetric quantification, HU-based densitometry, and standardized scoring systems within the same study were reported in a proportion of trials, reflecting a broader trend toward multidomain radiographic outcome assessment in contemporary SABG research.

### 3.6 Clinical assessment outcomes

Clinical outcomes have been variably reported and demonstrated substantial heterogeneity in both scope and methodology.

Across the 27 included studies, the clinical assessment of alveolar bone grafting (ABG) outcomes encompassed eight domains. The clinical parameters assessed across the included studies are summarized in Table 4.

**Table 4:** Clinical parameters assessed and complication occurrences.

Domain	Clinical Parameter Assessed	Studies Assessing Parameter (n)	Studies Assessing Parameter	Occurrence cases (n)	Study reporting occurrence
Fistula/Closure	Oronasal fistula presence	11	^7^^,^^8^^,^^12^^,^^17^^,^^20^^,^^22^, ^23^, ^24^^,^^26^^,^^27^^,^^30^	19	^24^^,^^26^^,^^30^
Wound Healing	Dehiscence	12	^8^^,^^12^^,^^14^^,^^15^^,^^17^^,^^18^^,^^20^^,^^22^^,^^23^^,^^25^^,^^26^^,^^29^	23	^8^^,^^12^^,^^14^^,^^18^^,^^20^^,^^23^^,^^25^^,^^26^^,^^29^
	Infection/surgical site infection	13	^8^^,^^13^, ^14^, ^15^^,^^17^^,^^18^^,^^20^, ^21^, ^22^, ^23^^,^^26^^,^^28^^,^^30^	12	^8^^,^^13^^,^^18^^,^^26^^,^^28^^,^^30^
	Pus discharge	1	^25^	0	-
	Tissue/flap necrosis	3	^7^^,^^17^^,^^20^	0	-
	Graft loss (clinical, partial or total)	5	^8^^,^^14^^,^^24^^,^^26^^,^^30^	23	^8^^,^^14^^,^^24^^,^^26^^,^^30^
	Wound healing duration (days to complete healing)	2	^25^^,^^28^	N/A	-
Periodontal	Pocket depth	2	^11^^,^^16^	N/A	-
	Gingival bleeding index	1	^11^	N/A	-
	Plaque index	1	^11^	N/A	-
Orthodontic	Tooth mobility (Miller classification)	1	^16^	N/A	-
	Tooth eruption (canine or other)	3	^13^^,^^18^^,^^27^	N/A	-
	Orthodontic/spontaneous tooth movement	2	^18^^,^^27^	N/A	-
	Arch alignment/dental arch stability	1	^27^	N/A	-
Functional/Structural	Alveolar arch continuity (clinical mobility test)	1	^24^	N/A	-
	Alveolar/maxillary ridge continuity	1	^27^	N/A	-
	Tooth bud/root injury	1	^7^	0	-
Pain/Symptoms	Swelling	2	^18^^,^^23^	N/A	-
	Temporary sensory complications (paresthesia/lip/chin numbness)	3	^7^^,^^17^^,^^18^	4	^7^^,^^18^
Aesthetic	Alar base symmetry	2	^18^^,^^27^	N/A	-
	Donor-site scar and contour satisfaction	3	^18^^,^^21^^,^^30^	2	^18^^,^^30^
Donor Site Morbidity	Gait disturbance/return to normal walking	6	^17^^,^^18^^,^^20^^,^^21^^,^^23^^,^^29^	5	^18^
	Hematoma at donor site	2	^21^^,^^29^	1	^29^
	Lateral femoral cutaneous nerve (LFCN) injury	1	^21^	3	^21^
	Overall donor-site morbidity (composite)	3	^18^^,^^21^^,^^30^	N/A	-
Other/Operational	Duration of surgery (minutes)	5	^17^^,^^18^^,^^20^^,^^21^^,^^29^	N/A	-
	Duration of hospital stay	1	^18^	N/A	-
	Cost of surgery	1	^18^	N/A	-

Surgical site infection was the most frequently assessed outcome in the reviewed studies, although not all findings were positive. This summary synthesizes the investigations from 13 studies ^8^^,^^13^, ^14^, ^15^^,^^17^^,^^18^^,^^20^, ^21^, ^22^, ^23^^,^^26^^,^^28^^,^^30^. Wound dehiscence, one of the most frequently tracked clinical parameters in this review, was assessed in 12 studies,^8^^,^^12^^,^^14^^,^^15^^,^^17^^,^^18^^,^^20^^,^^22^^,^^23^^,^^25^^,^^26^^,^^29^ while tissue or flap necrosis was assessed but absent in three studies.^7^^,^^17^^,^^20^ Oronasal fistula presence or closure was evaluated in 11 studies ^7^^,^^8^^,^^12^^,^^17^^,^^20^^,^^22^, ^23^, ^24^^,^^26^^,^^27^^,^^30^, assessed primarily through clinical inspection with fistula recurrence tracked in a minority. Periodontal parameters including pocket depth, gingival bleeding index, plaque index, and tooth mobility were assessed in only two studies,^11^^,^^16^ indicating limited attention to periodontal outcomes within the current ABG literature. Orthodontic outcomes such as canine eruption, spontaneous tooth movement, and alveolar arch continuity were each reported by only three studies,^13^^,^^18^^,^^27^ reflecting an evidence gap in functional dental integration following ABG.

Donor-site morbidity was a secondary but recurrent clinical concern across six studies that assessed gait disturbance or return to normal walking following iliac crest harvesting,^17^^,^^18^^,^^20^^,^^21^^,^^23^^,^^29^ with recovery ranging from one day to a mean of 9.5 days for conventional iliac crest procedures.^17^ Temporary sensory complications, including lip, chin, or lateral femoral cutaneous nerve paresthesia, were reported in three studies^7^^,^^17^^,^^18^ and resolved in all cases. Aesthetic outcomes, including alar base symmetry and donor-site scar appearance, were assessed in only one to three studies.^18^^,^^21^^,^^30^ Operational parameters such as surgery duration, hospital stay, and cost were also reported in a limited number of studies.^17^^,^^18^^,^^20^^,^^21^^,^^29^ Overall, clinical assessment methods were largely descriptive and based on direct clinical inspection, and no study used a standardized, validated composite clinical outcome instrument. This finding underscores the need for harmonized clinical reporting frameworks in future ABG trials.

### 3.7 Complications

Complications following ABG were reported in most included studies; however, the terminology, classification, and completeness of reporting were inconsistent. The most frequently reported recipient-site complication was wound dehiscence, documented in nine studies with a total of 23 cases across the included literature.^8^^,^^12^^,^^14^^,^^18^^,^^20^^,^^23^^,^^25^^,^^26^^,^^29^ Oronasal fistula was reported in 19 cases across three studies.^24^^,^^26^^,^^30^ Infection or surgical site infection was reported in six studies, accounting for 12 documented cases.^8^^,^^13^^,^^18^^,^^26^^,^^28^^,^^30^ Partial or total graft loss was reported in five studies, with a total of 23 cases.^8^^,^^14^^,^^24^^,^^26^^,^^30^ Tissue or flap necrosis was explicitly assessed in three studies, but no cases were reported.^7^^,^^17^^,^^20^ Pus discharge was evaluated in only one study, which reported no occurrences.^25^

Donor-site morbidity is a notable concern in studies involving iliac crest harvesting. Gait disturbance or delayed return to normal walking was assessed in six studies, with one study documenting five cases of prolonged gait impairment.^18^ Temporary sensory complications, including paresthesia or numbness of the chin or lower lip associated with chin symphysis harvesting, were reported in two studies, with four total cases across those reports.^7^^,^^18^ Lateral femoral cutaneous nerve (LFCN) injury was evaluated only by Lai et al.,^21^ who documented three cases in the inner cortico-cancellous iliac crest (ICIC) group and none in the outer cortico-cancellous iliac crest (OCIC) group. Donor-site hematoma was reported in one study, with one documented case.^29^ Donor-site scar dissatisfaction or hypertrophic scarring was noted in two studies.^18^^,^^30^ Overall, no study reported major vascular injury, permanent neurological deficit, or life-threatening complications, suggesting an acceptable safety profile for ABG procedures in the included populations. However, heterogeneity in complication reporting limits direct cross-study comparisons.

### 3.8 Follow-up durations

Follow-up duration varied considerably across the included studies, ranging from 3 month to more than 5 years, although most studies reported short-to mid-term follow-up. A 6-month follow-up was the most common assessment point, either as a single endpoint or as one of several time points.^8^^,^^9^^,^^11^^,^^12^^,^^15^^,^^19^^,^^21^^,^^22^^,^^27^^,^^30^^,^^31^

Several studies extended follow-up to 12 months,^7^^,^^13^^,^^17^^,^^23^^,^^25^ whereas others included intermediate time points such as 3 and 6 months.^5^^,^^14^^,^^16^^,^^24^ Short-term follow-up protocols were also reported, including assessments at 1 week, 1 month, and up to 4 months postoperatively.^20^^,^^28^

Only a limited number of studies reported longer follow-up periods, including 9 months^26^^,^^29^ and extended observation up to a mean of 5.25 years.^18^ Overall, the evidence base remains dominated by 6- to 12-month follow-up periods, with relatively few studies incorporating long-term evaluations.

## 4 Discussion

This scoping review mapped 27 randomized clinical trials evaluating graft materials and regenerative adjuncts in secondary alveolar bone grafting (SABG) published between 2016 and 2026. The evidence base is active and diverse; however, the dominant pattern is one of methodological heterogeneity and short follow-up duration, both of which limit definitive comparative conclusions in the literature. These findings reinforce the central role of autogenous iliac crest grafting in cleft rehabilitation, while also highlighting the ongoing and unresolved search for adjuncts and alternatives that can reliably preserve bone volume, reduce resorption, and minimize donor site morbidity.

### 4.1 Autogenous iliac crest graft

The mapped trials show that iliac crest–derived autogenous bone grafting remains the most common comparator in SABG and continues to function as the clinical reference standard because of its well-established biologic properties and longstanding clinical use.^32^ This pattern also suggests that most contemporary studies are not designed to replace iliac crest grafting outright, but rather to determine whether newer approaches can achieve comparable outcomes while reducing the treatment burden. In this review, donor-site morbidity emerged as a major driver of innovation, as postoperative pain, gait disturbance, and sensory complications demonstrate that the burden of treatment extends beyond the grafted cleft site, consistent with current evidence on donor-site morbidity.^4^ Many studies have therefore focused on reducing the adverse effects associated with harvesting rather than challenging the role of the graft itself.^5^^,^^24^ Importantly, the clinical impact of iliac crest grafting depends not only on graft biology but also on the harvesting technique. Evidence comparing minimally invasive and conventional approaches suggests meaningful differences in postoperative recovery, including hospital stay and opioid use.^33^

### 4.2 Biologic adjuncts and platelet concentrates

Platelet concentrates have been the most frequently investigated adjunctive strategy; however, the results have been inconsistent. One RCT found no significant difference in bone volume or density,^15^ whereas another study combining PRF with BMAC reported more favorable regenerative outcomes.^25^ This variability is both biologically plausible and methodologically expected, given the lack of standardized PRF preparation protocols.^15^^,^^25^ Differences in centrifugation speed and duration can alter leukocyte and platelet concentrations within the fibrin matrix, while variation in delivery methods, such as mixing, layering, or impregnation, may further influence biological and mechanical behavior.^34^ Broader evidence suggests that PRF can be used alone or in combination with other graft materials, and that combination approaches may enhance the regenerative environment.^35^^,^^36^ However, systematic review data indicate that PRF/PRP and control groups generally achieve similar outcomes in bone height, density, and volume, while also emphasizing substantial heterogeneity in outcome measurement.^37^

### 4.3 Cell-enriched regenerative strategies

The mapped trials demonstrate growing interest in cell-enriched strategies, particularly BMAC and SVF, as methods to enhance bone formation while preserving the structural role of the autogenous graft scaffold. Promising evidence from a 36-patient trial showed that combining BMAC with iliac cancellous bone improved bone volume, density, and reduced resorption at 12 months.^23^ However, this finding is not fully aligned with evidence synthesis, as one review and meta-analysis found no significant differences in bone volume or postoperative complications between stem cell–based approaches and conventional iliac crest grafting.^38^ Similarly, an SVF-supplemented graft trial reported comparable outcomes between groups and highlighted important limitations, including baseline imbalances and insufficient standardization in SVF characterization,^31^ despite broader literature suggesting that SVF has therapeutic potential across multiple fields through immunomodulatory, anti-inflammatory, and regenerative mechanisms.^39^ Tissue-engineered constructs, such as scaffolds seeded with mesenchymal stem cells, also produced outcomes comparable to autogenous grafts at longer follow-up,^14^ consistent with systematic review evidence showing similar cleft volume reduction between tissue-engineered bone substitutes and autogenous grafts, with the added benefit of avoiding donor-site morbidity.^40^ From a scoping perspective, these findings suggest that cell-based strategies represent a promising research direction; however, the current RCT landscape remains too limited and methodologically variable to support conclusions of superiority. At present, these approaches should be regarded as investigational adjuncts rather than established alternatives.

### 4.4 Barrier membranes and graft-substitute combinations

The mapped trials involving barrier membranes and graft-substitute combinations reflect a consistent clinical rationale: preserving space and limiting soft-tissue invasion may reduce early graft resorption and improve graft retention. Some RCT evidence supports this concept. For example, a study using guided bone regeneration with an acellular dermal matrix (ADM) membrane in combination with iliac grafting reported significantly reduced early resorption compared with grafting alone on CBCT volumetric assessment.^6^ This finding is consistent with prior evidence synthesis suggesting that covering iliac grafts with an acellular dermal matrix membrane may improve bone retention in unilateral cleft patients.^32^ Nevertheless, the overall evidence remains limited, particularly for long-term outcomes. From a scoping perspective, these findings indicate that barrier membranes and substitute materials are promising tools for improving graft preservation, but current evidence does not clearly establish superiority over standard approaches. Their value therefore appears to be context-dependent and should be interpreted considering radiographic outcomes, patient burden, and healthcare resource use.

### 4.5 Measurement heterogeneity

The mapped RCTs reveal substantial clinical and measurement heterogeneity, particularly in radiographic assessment methods. Studies used a wide range of endpoints, including volumetric fill, resorption rate, bone density, linear dimensions, and categorical scoring systems, together with diverse software platforms and analytic workflows. This variability is consistent with systematic review evidence identifying 3D CT/CBCT as the preferred approach for SABG evaluation while also emphasizing marked differences in protocols and reporting formats.^41^ Although three-dimensional imaging has improved quantitative assessment and is increasingly regarded as the preferred standard for future comparison, the lack of standardized evaluation frameworks continues to limit cross-study comparability.^42^^,^^43^ A particularly important methodological concern is the use of CBCT-derived bone density expressed as Hounsfield units. While some studies suggest a moderate positive correlation between CBCT gray values and CT-based Hounsfield units, available evidence indicates that CBCT gray values cannot be reliably converted into true Hounsfield units without calibration and standardized conversion procedures, because results are influenced by scanner characteristics, field of view, and region of interest.^44^ In addition, most trials focused on short-term imaging outcomes within 3–12 months, which do not fully capture clinically meaningful success such as graft stability during tooth eruption, orthodontic movement, and long-term remodeling. Outcome definitions also varied substantially. Existing evidence shows that criteria for graft success are inconsistent, and that factors such as age at grafting, oral hygiene, and tooth eruption status can influence reported results ^46^. From a scoping perspective, this heterogeneity is a major limitation of the current evidence base and underscores the need for standardized outcome measures, validated imaging protocols, and longer-term follow-up to improve both comparability and clinical relevance.

#### 4.5.1 Implications for clinical practice

The findings of this scoping review have important implications for clinical practice in SABG. First, iliac crest autogenous grafting remains the reference standard across more than a decade of contemporary randomized evidence, reaffirming that surgeons should regard this material as the benchmark against which all innovations are measured.

Second, adjunctive approaches may be considered when the clinical goal is to improve specific aspects of healing or bone regeneration, but their use should be tailored to the indication rather than adopted routinely. For patients in whom donor-site morbidity is a major concern, mandibular bone combined with adjunctive materials appears to perform comparably to iliac autogenous grafting while reducing gait disturbance and shortening operative time.

Third, advanced PRF formulations may improve wound healing and bone density at relatively low cost and risk, which may be particularly relevant in settings without access to cell-enriched technologies. However, clinicians should interpret these findings cautiously, as conventional PRF or PRP prepared using standard methods has not consistently demonstrated significant volumetric benefit.

Fourth, centers performing SABG should be encouraged to adopt CBCT-based volumetric assessment using standardized and calibrated software platforms, and to avoid reporting CBCT gray values as surrogate Hounsfield units without appropriate scanner calibration, as such measurements may misrepresent graft quality and confound interpretation.

### 4.6 Future research

Based on these findings, four priorities for future research can be identified. First, the cleft care community should develop a core outcome set for SABG that integrates volumetric, densitometric, categorical, and patient-reported endpoints, ideally modeled on similar initiatives in adjacent surgical fields. Second, CBCT-based density reporting should be standardized through scanner calibration and phantom-referenced gray-value conversion, replacing the current practice of uncalibrated HU comparisons. Third, biologic adjuncts, particularly platelet concentrates and BMAC, require harmonized preparation and characterization protocols before comparative effectiveness can be meaningfully established. Fourth, follow-up should routinely extend through canine eruption, orthodontic alignment, and definitive prosthodontic rehabilitation so that outcomes of greatest relevance to patients and families are captured. Multicenter pragmatic trials adopting these standards could help transform the field from a collection of small, heterogeneous single-center studies into a coherent and comparable evidence base capable of guiding practice changes.

### 4.7 Strengths and limitations

This scoping review has several methodological strengths. It provides a comprehensive and systematic mapping of RCT evidence in SABG over a 10-year period, capturing a broad range of graft materials, adjunctive strategies, and outcome measures. Restricting inclusion to randomized trials increases the methodological rigor of the mapped evidence base. The structured synthesis also enables the identification of major research patterns, inconsistencies, and knowledge gaps, and highlights methodological issues particularly variability in imaging protocols and outcome reporting that are often underemphasized in conventional systematic reviews. Preregistration with the Open Science Framework further supports transparency and reduces the risk of reporting bias.

Several limitations should also be acknowledged. The exclusion of non-English studies may have introduced language bias, particularly given the substantial volume of cleft surgery research conducted in non-English-speaking regions. Because this was a scoping review, no quantitative synthesis or effect size estimation was undertaken, and direct comparative effectiveness conclusions cannot be drawn. The included trials were highly heterogeneous with respect to graft composition, adjunct preparation protocols, defect morphology, surgical technique, follow-up duration, and outcome definitions, all of which limited cross-study comparability. Most trials also had modest sample sizes, often with fewer than 25 participants per arm, and relatively short follow-up, most commonly 6–12 months, without extending to canine eruption or orthodontic loading. Finally, the search was restricted to four electronic databases without supplementary hand-searching of reference lists or citation tracking, and the gray literature was not formally searched; publication bias favoring positive findings for novel adjuncts therefore cannot be excluded.

## 5 Conclusion

This scoping review of 27 randomized controlled trials confirms that autogenous iliac crest bone remained the most used graft material and the principal reference comparator throughout the 2016–2026 period, reinforcing its position as the clinical gold standard for SABG. None of the adjunctive or alternative approaches evaluated including platelet concentrates, cell-based therapies, barrier membranes, graft substitutes, or alternative donor sites demonstrated consistent superiority over autogenous grafting in key radiographic outcomes, including bone volume, density, and resorption. Most investigated innovations were designed to reduce donor-site morbidity and enhance early postoperative healing rather than to replace autogenous grafting. Among these, mandibular symphysis bone, advanced platelet-rich fibrin formulations, and bone marrow aspirate concentrate yielded the most promising outcomes. However, substantial heterogeneity in graft composition, imaging modalities, outcome definitions, and follow-up duration, together with the limited availability of long-term data extending through canine eruption and orthodontic loading, constrained cross-study comparability and weakened the strength of comparative conclusions.

## Patient's/Guardian's consent

Not applicable (this study is a scoping review of previously published studies; no individual patient data were collected or used).

## Author contributions

AME, HM, YYA, and AT contributed to this study according to their respective seniority and roles. Conceptualization and study design were performed by AT, YYA, HM, and AME. Database searching was conducted by AME, HM, and YYA, while full-text screening and data extraction were carried out by AME and HM. Interpretation of results and critical revision of the manuscript were undertaken by AT, YYA, and HM. AME drafted the manuscript. Final editing and revision were completed by AT, YYA, HM, and AME.

## Ethical approval

Not required (scoping review of published literature).

## Data availability

All data supporting this review are available within the manuscript. The review protocol is registered with the Open Science Framework (DOI: 10.17605/OSF.IO/WZQ86).

## Funding

No external funding was received for this study.

## Declaration of competing interest

The authors declare that they have no known competing financial interests or personal relationships that could have appeared to influence the work reported in this paper.
