# Supplementary material for: Graft materials and regenerative strategies in secondary alveolar bone grafting: A scoping review of randomized clinical trials
Source: J Oral Biol Craniofac Res. 2026 Aug 4;16(5):101508. doi: 10.1016/j.jobcr.2026.101508 (PMC13469950; doi:10.1016/j.jobcr.2026.101508)
Supplement: Supplementary file 1 [file mmc1.docx]

**Table S1. Database search strategy and record yield.**

| Search Engine | Boolean Operator | Search | Total |
| --- | --- | --- | --- |
| Pubmed | ((“Alveolar Process”[Mesh] OR “alveolar cleft”[tiab] OR “cleft alveolus”[tiab] OR “alveolar defect”[tiab] OR “alveolar defects”[tiab]) AND (“Cleft Lip”[Mesh] OR “Cleft Palate”[Mesh] OR cleft*[tiab] OR “cleft lip”[tiab] OR “cleft palate”[tiab]) AND (“Bone Transplantation”[Mesh] OR “bone graft*”[tiab] OR “alveolar bone graft*”[tiab] OR “secondary alveolar bone graft*”[tiab] OR SABG[tiab])) AND (randomized controlled trial[pt] OR controlled clinical trial[pt] OR random*[tiab] OR trial[tiab] OR placebo[tiab]) | 75 | 408 |
| Scopus | TITLE-ABS-KEY ( ( "alveolar cleft" OR "cleft alveolus" OR "alveolar defect*" OR "cleft alveolar" ) AND ( cleft* OR "cleft lip" OR "cleft palate" ) AND ( "bone graft*" OR "alveolar bone graft*" OR "secondary alveolar bone graft*" OR SABG ) AND ( random* OR trial OR placebo ) ) | 101 |  |
| Web of Science | TS=((“alveolar cleft” OR “cleft alveolus” OR “alveolar defect*” OR “cleft alveolar”) AND (cleft* OR “cleft lip” OR “cleft palate”) AND (“bone graft*” OR “alveolar bone graft*” OR “secondary alveolar bone graft*” OR SABG) AND (random* OR trial OR placebo)) | 117 |  |
| Embase | (('alveolar bone'/exp OR 'alveolar cleft':ti,ab,kw OR 'cleft alveolus':ti,ab,kw OR 'alveolar defect':ti,ab,kw OR 'alveolar defects':ti,ab,kw) AND ('cleft lip'/exp OR 'cleft palate'/exp OR 'cleft*':ti,ab,kw OR 'cleft lip':ti,ab,kw OR 'cleft palate':ti,ab,kw) AND ('bone transplantation'/exp OR 'bone graft*':ti,ab,kw OR 'alveolar bone graft*':ti,ab,kw OR 'secondary alveolar bone graft*':ti,ab,kw OR 'sabg':ti,ab,kw)) AND ('randomized controlled trial':it OR 'controlled clinical trial':it OR 'random*':ti,ab,kw OR 'trial':ti,ab,kw OR 'placebo':ti,ab,kw) | 115 |  |
